# Comparative efficacy of different therapeutic approaches in treatment naïve FLT3-mutated AML eligible for intensive chemotherapy: a Bayesian network meta-analysis of randomized trials

**DOI:** 10.1007/s00277-026-06948-8

**Published:** 2026-04-06

**Authors:** Antonella Bruzzese, Danilo Lofaro, Enrica Antonia Martino, Francesco Mendicino, Caterina Labanca, Santino Caserta, Eugenio Lucia, Virginia Olivito, Nicola Amodio, Fortunato Morabito, Ernesto Vigna, Massimo Gentile

**Affiliations:** 1 Hematology Unit, Department of Onco-Hematology, AO of Cosenza, Viale Della Repubblica Snc, 87100 Cosenza, Italy; 2 Department of Mathematics and Computer Science, University of Calabria, 87036 Rende, Italy; 3 Department of Experimental and Clinical Medicine, University of Catanzaro, Catanzaro, Italy; 4 AIL Sezione Di Cosenza, Cosenza, Italy; 5 Department of Pharmacy, Health and Nutritional Science, University of Calabria, Rende, Italy

**Keywords:** FLT3mutated (FLT3^mut^) AML, Treatment naïve AML, OS in AML, Meta-analysis

## Abstract

FLT3-mutated acute myeloid leukemia (FLT3mut AML) is associated with poor outcomes. Although FLT3 inhibitors (FLT3is) combined with chemotherapy improve responses, long-term survival remains limited, and the optimal first-line strategy is unclear. We conducted a Bayesian network meta-analysis of eight randomized trials, including 1,793 patients, to compare treatment used in patietne eligible for intensive chemoteherapy for overall survival (OS). Treatments studied were 3 + 7 with midostaurin, quizartinib, sorafenib, gemtuzumab ozogamicin (GO), glasdegib, CPX-351, and decitabine. FLT3i-based regimens improved outcomes but showed attenuated effects in this analysis, consistent with prior data. The small population with FLT3 mutation treated with GO + 3 + 7 and CPX-351 provided good outcomes (SUCRA 86.1% and 71.7%), while glasdegib + 3 + 7 and decitabine resulted in less effective strategies. Notably, 3 + 7 + GO showed superior benefit despite limited FLT3mut subgroup evidence. Ongoing studies are exploring CPX-351, GO, and FLT3i combinations, as well as novel strategies. Prospective, mutation-specific trials are needed to define optimal therapy.

**Supplementary Information:**

The online version contains supplementary material available at 10.1007/s00277-026-06948-8.

## Introduction

Acute myeloid leukemia (AML) is a biologically and clinically heterogeneous hematologic malignancy characterized by a spectrum of cytogenetic and molecular abnormalities. Among the most frequent genetic lesions (detected in approximately 25–30% of AML), there are mutations in the FMS-like tyrosine kinase 3 (FLT3) gene, the two most frequent ones are internal tandem duplications (FLT3-ITD) in the juxtamembrane domain, and point mutations or small deletions within the tyrosine kinase domain (FLT3-TKD) [1–5].

FLT3-ITD mutations have consistently been associated with an adverse prognosis, including higher relapse rates and reduced overall survival (OS), relapse-free survival (RFS), and event-free survival (EFS) compared to wild-type FLT3-ITD, particularly following chemotherapy or allogeneic stem cell transplantation (alloSCT) [1, 2, 6–9]. In contrast, the prognostic impact of FLT3-TKD mutations remains less well-defined and may be influenced by co-occurring genetic abnormalities.

The therapeutic landscape for FLT3-mutated (FLT3^mut^) has evolved substantially with the introduction of FLT3 inhibitors (FLT3is). The pivotal phase III RATIFY trial demonstrated that the addition of midostaurin to standard induction and consolidation chemotherapy significantly improved complete remission (CR) rates, OS, and RFS with the addition of midostaurin to conventional chemotherapy in newly diagnosed, fit patients with FLT3^mut^ AML [10]. As a result, given the therapeutic relevance of FLT3 targeting, current European Leukemia Net (ELN) and the National Comprehensive Cancer Network (NCCN) guidelines recommend FLT3 mutation ing at diagnosis in all AML patients, and recommend adding midostaurin to intensive chemotherapy [11, 12].

Other FLT3is have also been evaluated in both pre-alloSCT and post-alloSCT settings. Sorafenib has been investigated in phase II trials with conflicting results when added to standard chemotherapy [13, 14]. More recently, the phase III QuANTUM-First trial showed that quizartinib, when combined with intensive chemotherapy, led to improved CR rates, OS, and EFS compared to chemotherapy alone in patients with FLT3-ITD AML [15].

In parallel with the development of FLT3is, several other agents have been explored in the frontline treatment of newly diagnosed AML irrespective of FLT3^mut^ [16–21].

While several of these studies included patients with FLT3 mutations, most were not specifically powered to assess efficacy in this subgroup. Furthermore, no head-to-head randomized trials have directly compared different FLT3is combined with chemotherapy, nor have studies systematically evaluated the addition of FLT3is to alternative intensive regimens.

The present network meta-analysis aims to synthesize available evidence on the comparative efficacy of intensive strategies in newly diagnosed, fit patients with FLT3^mut^ AML. By integrating data from randomized trials, this analysis seeks to inform optimal first-line therapeutic approaches in this molecularly defined subgroup.

## Methods

### Search strategy

A systematic literature review was conducted to identify randomized controlled trials (RCTs) evaluating first-line treatments in newly diagnosed (treatment-naïve, TN) AML patients harboring FLT3 mutations fit for intensive chemotherapy. Both published and unpublished studies, including congress abstracts, were considered eligible provided that they reported sufficient methodological and outcome data.

### Eligibility criteria

Studies were eligible for inclusion if they met the following criteria: (i) randomized controlled design, regardless of blinding status; (ii) enrolled adult patients with TN FLT3^mut^ AML; (iii) investigated novel or non-standard first-line regimens in the experimental arms, with standard induction therapy as comparator; and (iv) reported at least one efficacy outcome, including OS, specifically for the FLT3-mutated subgroup.

Studies were excluded if they were non-randomized or non-prospective, lacked relevant outcome data for the FLT3-mutated subgroup, had unclear or incomplete methodology, or evaluated treatments outside the first-line.

### Data extraction and quality assessment

Two reviewers (A.B. and M.G.) independently screened titles and abstracts, assessed full-text articles for eligibility, and extracted data using a standardized form.

Extracted data included: i) study identifiers (first author, publication year); study design characteristics, patient demographics, patient FLT3 mutation status, treatment details, and reported hazard ratios (HRs) for OS in the FLT3-mutated population. Each reviewer maintained a separate database; results were compared, and discrepancies were resolved by discussion. Duplicates were removed following cross-checking.

### Risk of bias assessment

The risk of bias for included studies was evaluated using the Cochrane Risk of Bias 2 (RoB 2) tool [22], which assesses bias across the five domains: randomisation process, deviations from intended interventions, missing outcome data, measurement of outcomes, and selection of the reported result. Each study was independently assessed by two reviewers. Any disagreements were resolved through discussion or consultation with a third reviewer, if necessary. Studies were classified as having ‘Low risk’, ‘Some concerns’, or’High risk’ of bias. Summary results were visualized using traffic‑light plots and a bar chart.

### Statistical analysis

A Bayesian random‑effects contrast‑based network meta‑analysis (NMA) was performed to compare the efficacy of different first-line regimens in FLT3^mut^ AML. The outcome analysed was the HR of the OS contrast. Non-informative normal priors (mean 0, large variance) were applied to basic treatment effects, while a wide Uniform prior was used for the between-study standard deviation (τ). Robustness was assessed using a moderately informative half-Normal prior for τ.

Four parallel Markov chains were run (40,000 iterations; burn-in = 20,000; thinning = 10). Convergence was confirmed visually and with Brooks-Gelman-Rubin diagnostics (all potential scale-reduction factors ≤ 1.05). Model adequacy was judged by the posterior mean residual deviance and the deviance-information criterion (DIC). A forest plot was used to display pooled posterior HRs and 95% credible intervals (CrIs) for each regimen versus the reference regimen.

Treatment ranking was summarised with surface under the cumulative ranking curve (SUCRA) and reported alongside posterior medians and 95% CrIs for every pairwise contrast.

Sensitivity analyses were carried out to assess robustness of the results, in particular: i) an NMA limited to trials that enrolled only FLT3-mutated patients (excluding subgroup data); ii) leave-one-treatment-out (NMAs leaving out one treatment each time) analyses to eventually identify influential studies; iii) Bayesian meta-regressions incorporating median age, an indicator for “older-patient-only” trials and an indicator of subgroups derived results, to explore potential effect modification and violations of NMA assumptions.

All analyses were conducted in R 4.5.0 [23], with NMA models implemented in JAGS, via the gemtc [24] and rjags R packages [25].

The present network meta-analysis was not registered in the PROSPERO international prospective register of systematic reviews.

## Results

### Study selection

**Fig. 1:**
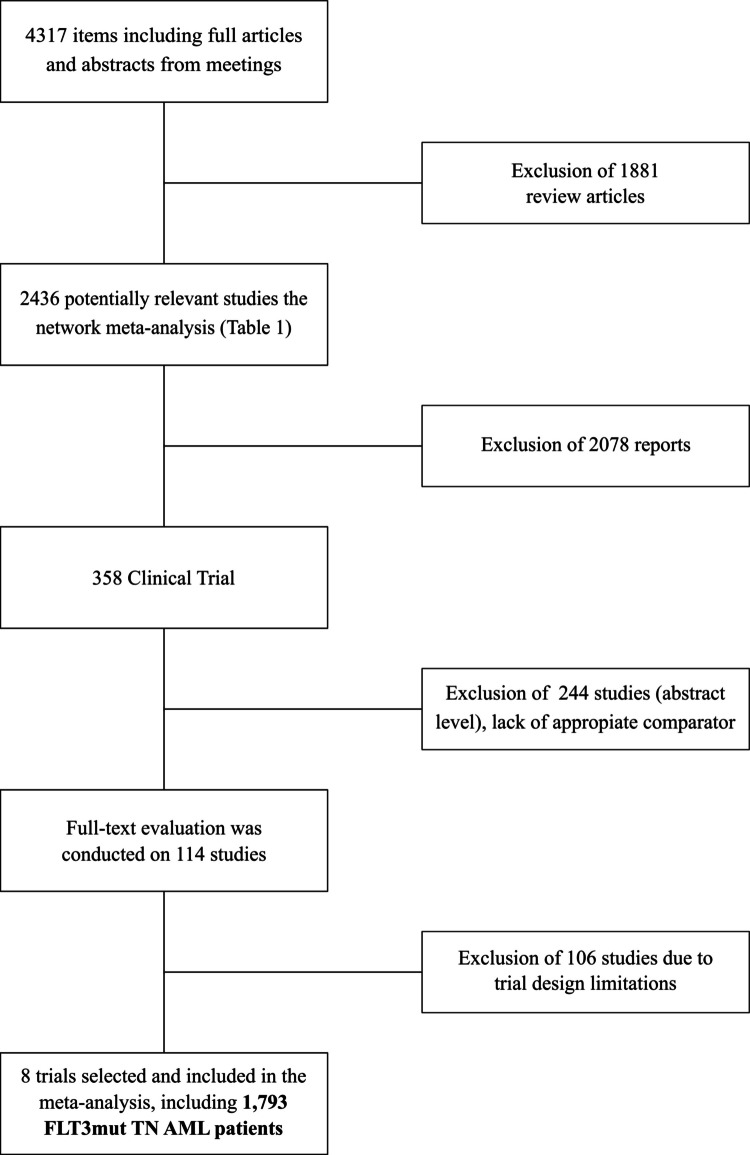
PRISMA 2020 flow diagram of study selection

The study selection process is summarized in the PRISMA flow diagram (Fig. 1).

From an initial pool of 4,317 records, identified between 2012 and 2025—including full articles and conference abstracts—1,881 review articles were excluded, yielding 2,436 potentially eligible studies. After title and abstract screening, 2,078 reports were excluded due to irrelevance or ineligible design, leaving 358 clinical trials for further evaluation. Among these, 244 studies were excluded owing to the absence of a suitable comparator arm. Full-text review was conducted on 114 studies, of which 106 were excluded for methodological limitations or lack of data specific to FLT3-mutated AM. Ultimately, eight trials, including 1,793 TN FLT3^mut^ AML patients, were deemed eligible for inclusion in the network meta-analysis (Table 1).

**Table 1:** Main characteristics of clinical trials included in the meta-analysis

Trial (Authors)	Publication year	Treatment arms	Randomized patients	FLT3-mutated
RATIFY (Stone et al.)	2017	Midostaurin + 3 + 7 3 + 7	360 357	360 357
QuANTUM First (Erba et al.)	2023	Quizartinib + 3 + 7 3 + 7	268 271	268 271
SORAML (Rolling et al.)	2021	Sorafenib + 3 + 7 3 + 7	134 133	23 23
Study by ALLG (Loo et al.)	2023	Sorafenib + 3 + 7 3 + 7	35 33	35 33
ALFA-0701 (Castaigne et al.)	2012	GO + 3 + 7 3 + 7	140 140	22 27
NCT02172872 (Lübbert et al.)	2023	10 day-DEC 3 + 7	303 303	32 22
BRIGHT AML 1019 (Sekeres et al.)	2023	Glasdegib + 3 + 7 3 + 7	201 203	12 14
Study 301 (Lancet et al.)	2021	CPX-351 3 + 7	153 156	22 21

Abbreviations: *GO* gentuzumab + ozogmamicin, *DEC* - decitabin

### Risk of bias assessment

**Fig. 2:**
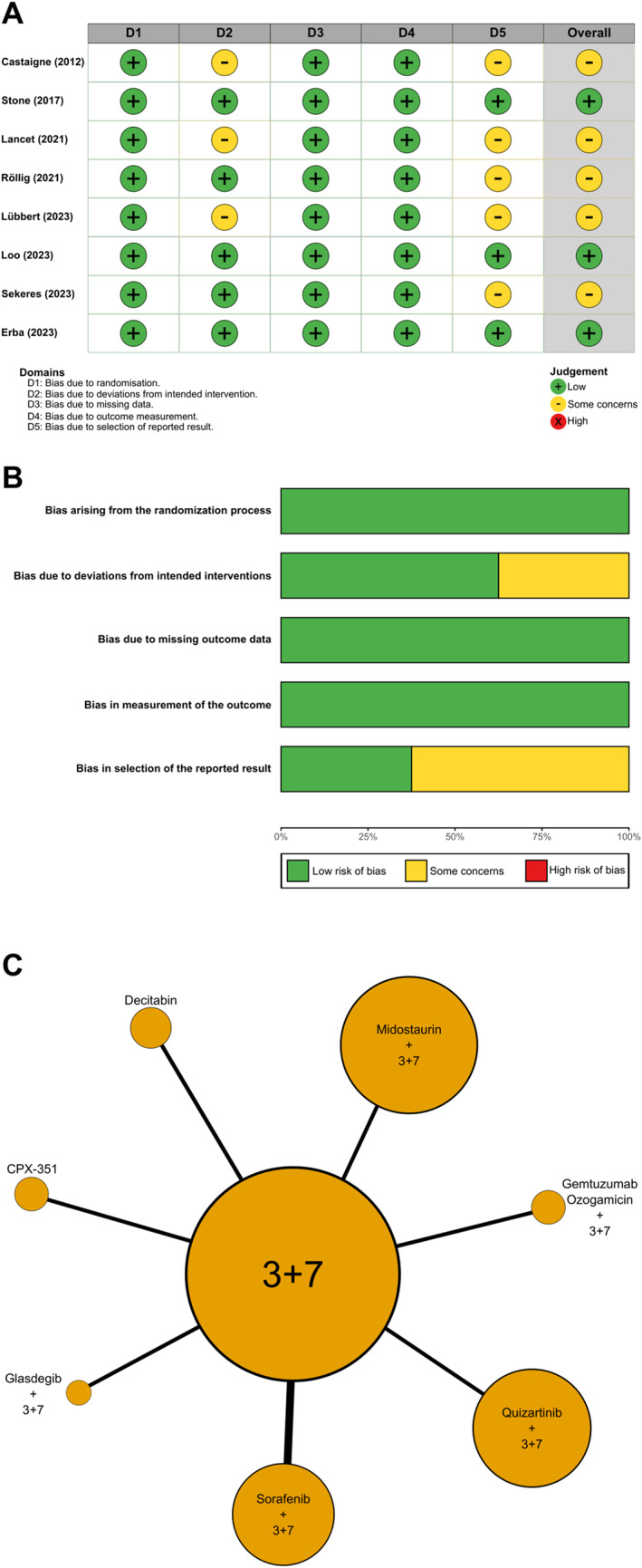
Risk-of-bias assessment of the included RCTs. (**A**) Traffic-light plot of domain-level RoB 2 ratings. (**B**) A stacked bar chart summarising the proportion of trials rated Low risk (green), Some concerns (yellow), or High risk (red) across each domain. Evidence network for overall survival (**C**). Nodes represent treatment regimens, scaled to the total number of FLT3^mut^ participants; edges represent direct trial comparisons, with line thickness proportional to the number of trials

Risk of bias was assessed for all included studies using the Cochrane RoB2 tool (Fig. 2). Nine randomised controlled trials encompassing eight distinct treatment regimens were included in the network meta-analysis.

Overall, three trials were judged to be at low risk of bias, while five of the remaining were rated as having some concerns; no study was classified as being at high risk. The most frequent source of concern related to Domain 2 (deviations from intended interventions) is primarily due to open-label study designs. However, given that overall survival was the primary outcome, these deviations were unlikely to materially influence effect estimates.

Additional concerns were identified in Domain 5 (selection of the reported result) for five trials, as HR estimates for overall survival were derived from subgroup analyses of FLT3-mutated patients that were not pre-specified in the original study protocols. This raised the potential for selective outcome reporting, although no clear evidence of systematic reporting bias was observed.

### Network meta-analysis of overall survival

The evidence network included eight strategies configured in a star-shaped topology, with standard 3 + 7 induction chemotherapy as the common comparator (Fig. 2). Direct comparisons were available only between each experimental regimen and 3 + 7; no closed loops were present, precluding statistical assessment of inconsistency. Between-study heterogeneity was moderate, with a posterior median between-study standard deviation (τ) of 0.43 (95%CrI 0.02–1.14, log-HR scale). Overall model fit was acceptable (DIC = 15.0).

### Comparative efficacy versus standard induction (3 + 7)

**Fig. 3:**
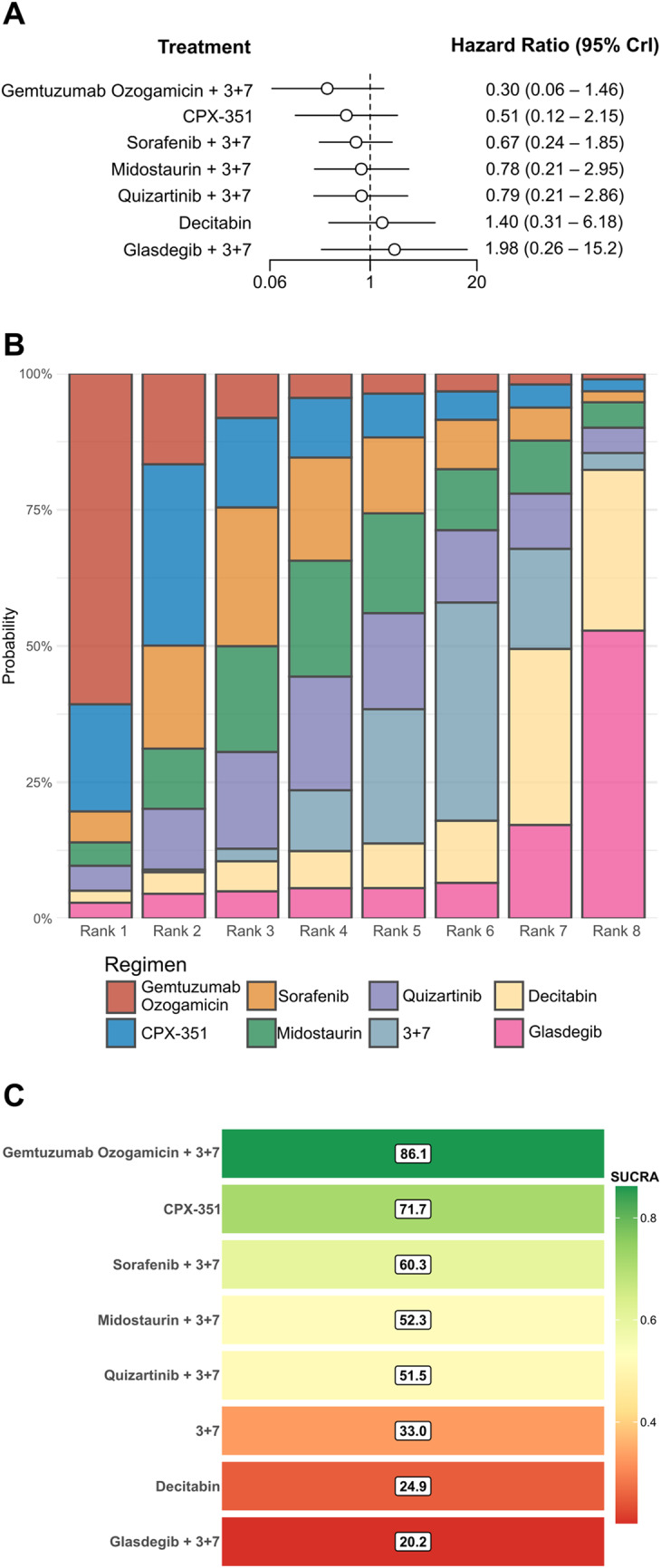
Bayesian forest plot of HRs *versus* standard 3 + 7 (**A**). Posterior median HRs and 95% CrIs from the random-effects NMA are plotted for each experimental regimen against the common comparator 3 + 7. Treatments are ordered from most to least favourable (left to right). The dashed vertical line marks HR = 1 (no difference). Ranking of analysed regimens by SUCRA values. (**B**) Stacked rankogram showing the posterior probability that each regimen occupies every rank position (Rank 1 = best, Rank 8 = worst). (**C**) Heat-map of SUCRA values. Numbers inside bars give SUCRA expressed as percentages

Figure 3 presents posterior median HRs and 95% CrIs for each experimental treatment *versus* standard 3 + 7.

Despite the small population size of FLT3^mut^ patients include in the trail, the combination of GO with 3 + 7 showed the most favourable point estimate (HR = 0.30, 95%CrI 0.06–1.46), followed by CPX-351 (0.51, 0.12–2.15) and Sorafenib + 3 + 7 (0.67, 0.24–1.85). Combinations involving Midostaurin and Quizartinib yielded HRs clustered around 0.8. In contrast, Decitabine and Glasdegib + 3 + 7 were associated with numerically higher HRs (1.40 and 1.98, respectively), suggesting a trend toward inferior outcomes relative to the control. However, all CrIs overlapped the null value (HR = 1), reflecting substantial uncertainty, largely attributable to the reliance on subgroup-level data for five of the seven experimental regimens.

### Treatment ranking

Treatment ranking based on SUCRA values is illustrated in Fig. 3. SUCRA-based ranking of analysed regimens was as follow: (1) GO + 3 + 7 regimen (SUCRA 86.1%), followed by CPX-351 (71.7%), Sorafenib + 3 + 7 (60.3%), Midostaurin + 3 + 7 (52.3%), and Quizartinib + 3 + 7 (51.5%). These data must interpreted with caution, particularly for GO plus 3 + 7 and CPX-351, given the small number of FLT3-mutated patients contributing to these treatment nodes. Standard 3 + 7 ranked in the lower half of the distribution (SUCRA 33.0%), whereas Decitabine and Glasdegib plus 3 + 7 had the weakest probabilities of benefit (SUCRA 24.9% and 20.2%, respectively).

### Pairwise contrasts

All 28 indirect pairwise contrasts between treatments were directionally consistent with the SUCRA rankings, but lacked statistical significance. CrI for all comparisons crossed unity, reflecting wide uncertainty intervals (Table 2). These findings underscore a key limitation of the network’s star-shaped configuration, wherein all regimens are compared only to the common comparator (3 + 7), with no direct head-to-head trials between experimental treatments.

**Table 2:** Pairwise relative treatment effects comparison as HR (95% CrI)

**Gemtuzumab** **Ozogamicin**							
0.57 (0.07–5.24)	**CPX-351**						
0.44 (0.06–2.87)	0.76 (0.14–4.33)	**Sorafenib**					
0.38 (0.05–2.86)	0.67 (0.09–4.21)	0.87 (0.16–4.35)	**Midostaurin**				
0.38 (0.05–2.72)	0.66 (0.09–4.58)	0.87 (0.17–4.36)	0.99 (0.16–6.32)	**Quizartinib**			
0.30 (0.06–1.46)	0.52 (0.12–2.15)	0.68 (0.24–1.85)	0.78 (0.21–2.95)	0.78 (0.21–2.86)	**3 + 7**		
0.21 (0.02–2.03)	0.37 (0.04–3.06)	0.48 (0.08–3.02)	0.56 (0.08–4.08)	0.56 (0.08–4.12)	0.72 (0.16–3.19)	**Decitabin**	
0.15 (0.01–1.95)	0.26 (0.02–3.20)	0.34 (0.04–3.38)	0.40 (0.04–4.49)	0.40 (0.04–4.73)	0.52 (0.07–3.92)	0.71 (0.05–9.01)	**Glasdegib**

### Sensitivity analyses

Multiple sensitivity analyses were undertaken to assess the robustness of the results and to identify potential violations of model assumptions.

First, restricting the analysis to trials exclusively enrolling FLT3^mut^ patients [12, 14, 15] yielded consistent SUCRA rankings (Supplementary Tables 1, 2), with 3 + 7 consistently ranked lowest. Effect sizes from the original trials showing statistically significant OS benefit (e.g., RATIFY and QuANTUM-First) were attenuated in the network model. While point estimates remained similar (HR = 0.78), the 95% CrIs widened and crossed unity in both cases: Midostaurin (HR = 0.78; 95% CrI, 0.52–3.00) and Quizartinib (HR = 0.78; 95% CrI, 0.52–1.17).

Leave-one-treatment-out analyses demonstrated minimal impact on SUCRA rankings and pairwise HRs (Supplementary Figs. 1 and 2). Removal of any single treatment did not alter the top three ranked therapies, and the absolute percentage change in HRs did not exceed ~ 3%.

In addition, we performed a dedicated sensitivity analysis excluding decitabine from the network.

After removal of decitabine, the relative positioning of the main regimens of interest remained substantially unchanged. The corresponding SUCRA profiles showed only minor redistribution across adjacent ranks, without inversion of the relative ordering of top-ranked treatments (Supplementary Table 3).

Consistently, the pairwise HR estimates involving 3 + 7 + GO and CPX-351 remained stable, with differences well within the range observed in the leave-one-treatment-out analyses (absolute changes < 3%).

### Meta-regression analyses

Exploratory meta-regression analyses were conducted to explore the potential influence of study-level covariates (Supplementary Table 4). Incorporating median age as a continuous covariate did not meaningfully modify the treatment effect estimated (HR ratio = 0.99 per 10-year increase; 95% CrI 0.02–21.18). Model fit improved only marginally (DIC = 13.4 *versus* 15.0), and τ remained stable. Similarly, the introduction of a binary covariate for trials restricted to older populations yielded null findings.

A third model assessing whether dedicated FLT3^mut^ trials differed from subgroup-based analyses also showed no significant effect modification (HR ratio = 0.89; 95% CrI, 0.19–6.12), although this model introduced slightly greater residual heterogeneity (τ = 0.55) and a modest increase in DIC (15.6). These findings suggest that variation in patient age and trial design does not significantly confound treatment effect estimates within the network.

## Discussion

For nearly four decades, the combination of cytarabine and an anthracycline (commonly referred to as the 3 + 7 regimen) has represented the standard of care for patients with AML eligible for intensive chemotherapy. However, growing insights into the genetic and molecular heterogeneity of AML have facilitated a shift toward more personalized treatment approaches. This shift is reflected in recent classifications, such as the International Consensus Classification (ICC), which underscore the complexity of AML subtyping and highlight the challenges of individualized treatment selection [1].

In the subset of AML patients harboring FLT3^mut^, a group comprising approximately 25–30% of adult AML, therapeutic advances have been largely driven by the development of FLT3is. These agents have shown efficacy both as monotherapies and in combination with intensive regimens. Despite this progress, outcomes remain suboptimal, with median RFS around 30 months, and CR rates approximately 70% in clinical trials, suggesting that FLT3i-based triplet regimens may still be improved [10, 15].

In the present NMA, when restricted to trials enrolling exclusively FLT3^mut^ patients treated with 3 + 7 plus a FLT3i, no substantial differences in efficacy emerged among the experimental regimens. SUCRA values were comparable, and standard 3 + 7 consistently ranked lowest, suggesting a reduced probability of being the most effective regimen. Notably, effect sizes from pivotal trials, such as RATIFY (midostaurin) and QuANTUM-First (quizartinib), which had reported statistically significant improvements in OS, were attenuated in the NMA [10, 14, 15].

This attenuation reflects the application of a random-effects prior, which accounts for between-study heterogeneity and incorporates hierarchical shrinkage, thereby widening credible intervals when few studies inform a comparison. These findings are consistent with a prior meta-analysis by which similarly found no significant OS differences among FLT3is [26]. A retrospective analysis also suggested improved RFS and OS with FLT3is, particularly in lower-intensity treatment settings [27].

Emerging FLT3is, such as crenolanib, may offer further therapeutic gains [28].

Despite the important limitations of our star-shaped network—namely, the absence of direct head-to-head comparisons between experimental regimens and the small sample sizes of the included trials—the network meta-analysis did not identify statistically significant differences among treatments. Although 3 + 7 plus GO and CPX-351 emerged as the highest-ranked regimens, these findings should be interpreted strictly as hypothesis-generating and do not constitute robust evidence of clinical superiority. Most patients with FLT3^mut^ AML fall into the intermediate-risk category benefiting and may therefore derive benefit from GO [16, 17], while a subset with secondary AML may be considered candidates for CPX-351.

Importantly, no randomized trial has specifically evaluated these regimens in FLT3^mut^ AML population, despite a plausible biological and clinical rationale.

In the ALFA-0701 trial, the 49 FLT3-mutated patients enrolled did not show substantial differences in performance status or cytogenetic risk compared with the patients included in the other trials analyzed in the present meta-analysis [16, 17]. More recently, a small retrospective study including 11 FLT3^mut^ patients reported a CR rate of 91% with the combination of GO, midostaurin, and intensive chemotherapy (Weinbergerová et al. [29]). Consistent with these findings, the SAL-MODULE phase I study investigated a four-drug regimen (3 + 7 plus GO and midostaurin) in 12 patients, 10 of whom harbored FLT3 mutations, achieving a CR rate of 75% and an overall response rate (ORR) of 92% [30].

By contrast, another phase I study conducted in a similar patient population reported a composite CR rate of 76% with OS rates of 79%, 65%, and 39% at 6 months, 1 year, and 2 years, respectively, and a high incidence of grade ≥ 3 cytopenias [31].

Further support for a potential sensitivity to GO in FLT3^mut^ AML comes from the UK NCRI AML19 trial, in which the addition of GO to FLAG-Ida (fludarabine, cytarabine, G-CSF, idarubicin) was associated with improved outcomes in younger patients with FLT3^mut^ AML, including reduced relapse rate and improved long-term survival, especially among those with co-occurring NPM1 and FLT3 mutations [32].

Post hoc analyses performed on patients enrolled in the 301 study revealed increased sensitivity of FLT3-ITD blasts to CPX-351, including enhanced drug uptake and cytotoxicity compared to FLT3-wild-type blasts [33].

Although robust data are lacking on combining CPX-351 with FLT3is, preliminary results are encouraging. A phase I study of CPX-351 + gilteritinib in relapsed/refractory AML reported a composite CR/CRi rate of 46.2% and median OS of 11.9 months, with manageable toxicity [34]. The phase Ib V-FAST trial explored CPX-351 + midostaurin in newly diagnosed FLT3^mut^ AML, showing CR rates of 82% (FLT3-ITD) and 83% (FLT3-TKD) [35]. Several ongoing trials are evaluating combinations of CPX-351, GO, and FLT3is. One such phase III trial (NCT04293562) is randomizing newly diagnosed AML patients to receive chemotherapy with or without GO, with the addition of gilteritinib for FLT3-mutated patients.

Beyond the small sample size of FLT3^mut^ patients enrolled in most of the analysed trials, another important limitation of the present study are lack of prospective stratification by FLT3 status and the lack of data on alloSCT. Among the analyzed trials, the exact percentage of patients that received alloSCT and their outcomes are available only for patients enrolled in the ALFA 0701 and QUANTUM-first; for the other studies, data are incomplete. Moreover, comparing the percentage of transplanted patients in ALFA 0701 and QUANTUM-first, it is notable that only 1/3 of patients in ALFA0701 underwent alloSCT compared to 50% of QUANTUM-first, reflecting the different approaches to alloSCT in AML patients in a 10 year period [15–17]. Moreover, data on therapeutic options for post-transplant relapses remain limited, as comparative evidence on different consolidation strategies—such as alloSCT versus FLT3inhibitor–based approaches—is lacking. Similarly, information on post-relapse therapies is sparse, and both alloSCT rates and post-relapse treatment strategies may substantially influence OS outcomes.

Another important limitation to take into consideration is that our NMA focuses on OS that is influenced not only by the efficacy and safety of chemotherapy but also by the optimization of supportive care and possible post relapse salvage strategies, whose data are not comparable in the selected studies.

FLT3is are also being explored in less intensive regimens. Preclinical synergy between FLT3is and BCL-2 inhibitors has led to studies combining venetoclax with FLT3is, yielding promising early results and potentially more durable responses [36–38]. Although decitabine did not improve outcomes *versus* standard induction in FLT3^mut^ AML [19], further research is needed to evaluate whether FLT3i combinations with hypomethylating agents may enhance therapeutic response.

In conclusion, FLT3^mut^ AML represents a biologically distinct and therapeutically challenging subset of the disease. This network meta-analysis suggests that 3 + 7 + GO and CPX-351 may be effective in the treatment of FLT3^mut^ patients; however, definitive conclusions are limited by small sample sizes and a lack of direct comparative trials. Future studies should focus on integrating FLT3is with additional targeted agents and optimizing treatment intensity based on individual molecular and clinical profiles. The continued development of rational combinations tailored to FLT3^mut^AML has the potential to significantly improve long-term outcomes in this high-risk population.

## Supplementary Information

Below is the link to the electronic supplementary material.

Supplementary file 1 (DOCX 20 KB)

Supplementary file 2 (PNG 75.5 KB)

Supplementary file 3 (TIFF 257 KB)

Supplementary file 2 (PNG 150 KB)

Supplementary file3 (TIFF 369 KB)

## Data Availability

Data sharing does not apply to this article, as no new data were generated or analyzed in this study.
