# Supplementary material for: Comparative efficacy of different therapeutic approaches in treatment naïve FLT3-mutated AML eligible for intensive chemotherapy: a Bayesian network meta-analysis of randomized trials
Source: Ann Hematol. 2026 Apr 6;105(5):226. doi: 10.1007/s00277-026-06948-8 (PMC13053374; doi:10.1007/s00277-026-06948-8)
Supplement: Supplementary file 1 — (DOCX 20 KB) [file 277_2026_6948_MOESM1_ESM.docx]

**SUPPLEMENTARY APPENDIX CONTENTS**

**Comparative Efficacy of Intensive Frontline Regimens in FLT3-Mutated AML: A Bayesian Network Meta-Analysis of Randomized Trials**

Antonella Bruzzese^1^, Danilo Lofaro^2^, Enrica Antonia Martino^1^, Francesco Mendicino^1^, Caterina Labanca^1^, Santino Caserta^1^, Eugenio Lucia^1^, Virginia Olivito^1^, Nicola Amodio^3^, Fortunato Morabito^4*^, Ernesto Vigna^1*^, Massimo Gentile^1,5*^.

^1^Hematology Unit, Department of Onco-Hematology, AO of Cosenza, Cosenza, Italy; ^2^Department of Mathematics and Computer Science, University of Calabria, 87036 Rende, Italy; ^3^Department of Experimental and Clinical Medicine, University of Catanzaro, Catanzaro, Italy; ^4^AIL Sezione di Cosenza, Italy; ^5^Department of Pharmacy, Health and Nutritional Science, University of Calabria, Rende, Italy

* These authors contributed equally as senior authors.

**Correspondence**: Massimo Gentile, MD, Hematology Unit, AO of Cosenza, Italy; 87100 Cosenza, Italy; viale della Repubblica snc, e-mail: massimo.gentile@unical.it; ph: +39-0984-681329; fax: +39-0984-681329; Ernesto Vigna, MD, Hematology Unit, AO of Cosenza, Italy; 87100 Cosenza, Italy; viale della Repubblica snc, e-mail:ernesto.vigna@aocs.it; ph: +39-0984-681329; fax: +39-0984-681329.

**Keywords:** FLT3mutated (FLT3^mut^) AML, treatment naïve AML, OS in AML, meta-analysis

**Supplementary Tables:** 3; **Supplementary Figures**: 2.

**Supplementary Table 1.** SUCRA values from NMA restricted to trials enrolling only FLT3^mut^ patients

| **Treatment** | **SUCRA** |
| --- | --- |
| Midostaurin | 0.631 |
| Quizartinib | 0.624 |
| Sorafenib | 0.618 |
| 3+7 | 0.126 |

**Supplementary Table 2.** Pairwise comparison from NMA restricted to trials enrolling only FLT3^mut^ patients.

| **Midostaurin** |  |  |  |
| --- | --- | --- | --- |
| 0.99 (0.57-1.75) | **Quizartinib** |  |  |
| 1.03 (0.46-2.21) | 1.03 (0.47-2.28) | **Sorafenib** |  |
| 0.78 (0.52-1.12) | 0.78 (0.52-1.17) | 1.32 (0.68-2.53) | **3+7** |

**Supplementary Table 3**: Rankogram, after removal of decitabine,

| Treatment | Rank | | | | | | |
| --- | --- | --- | --- | --- | --- | --- | --- |
|  | 1 | 2 | 3 | 4 | 5 | 6 | 7 |
| GO | 0.606 | 0.173 | 0.09 | 0.047 | 0.0375 | 0.0308 | 0.0155 |
| CPX | 0.2 | 0.347 | 0.173 | 0.102 | 0.0802 | 0.0632 | 0.0342 |
| Sora | 0.0672 | 0.189 | 0.252 | 0.207 | 0.137 | 0.107 | 0.0402 |
| Mido | 0.054 | 0.116 | 0.196 | 0.222 | 0.189 | 0.147 | 0.0755 |
| Quiz | 0.045 | 0.115 | 0.188 | 0.218 | 0.195 | 0.156 | 0.084 |
| 3+7 | 0.00025 | 0.00625 | 0.0378 | 0.144 | 0.297 | 0.408 | 0.107 |
| Gla | 0.027 | 0.0542 | 0.0625 | 0.0602 | 0.0638 | 0.0888 | 0.644 |

**Supplementary Table 4. Bayesian meta-regression sensitivity analyses of study-level covariates.** HR ratio and their 95% CrI, the residual between-study heterogeneity (τ), and the deviance-information criterion (DIC).

| **Covariate** | **Comparison** | **HR ratio** | **95 % CrI** | **τ** | **DIC** |
| --- | --- | --- | --- | --- | --- |
| Median study age | Numeric (values per 10-year increase) | 0.987 | 0.016-21.179 | 0.43 | 13.4 |
| Elderly-only study | Elder-only vs. not age-restricted studies | 1.033 | 0.001-370.145 | 0.42 | 14.95 |
| Dedicated trial vs subgroup | Subgroup vs dedicated studies results | 0.895 | 0.191-6.122 | 0.55 | 15.56 |

**Supplementary Figure Legends**

**Supplementary Figure S1.** Stability of treatment ranking in leave-one-treatment-out analyses. Box plots show the SUCRA values distribution in the seven sequential NMAs, excluding one regimen each time. Red points represent base SUCRA values.

**Supplementary Figure S2.** Absolute percentage change in posterior HRs after leave-one-treatment-out analyses. Triangular heat-map matrix showing the absolute % change in every pairwise posterior HR when each regimen is omitted in turn.
