# Supplementary figures and images for: Comparative efficacy of different therapeutic approaches in treatment naïve FLT3-mutated AML eligible for intensive chemotherapy: a Bayesian network meta-analysis of randomized trials
Source: Ann Hematol. 2026 Apr 6;105(5):226. doi: 10.1007/s00277-026-06948-8 (PMC13053374; doi:10.1007/s00277-026-06948-8)

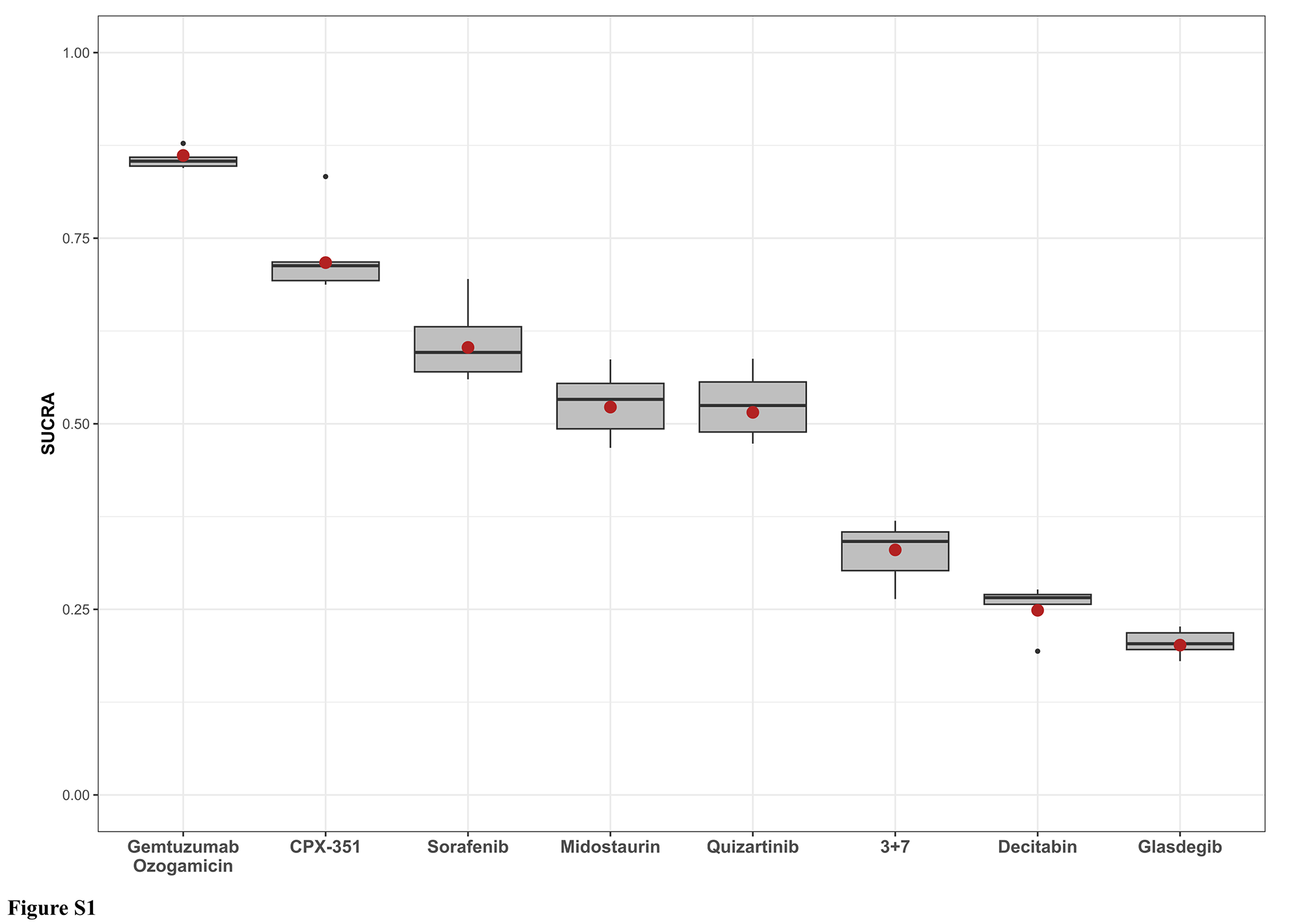

Supplement: Supplementary file 2 — (PNG 75.5 KB) [file 277_2026_6948_Fig4_ESM.png]

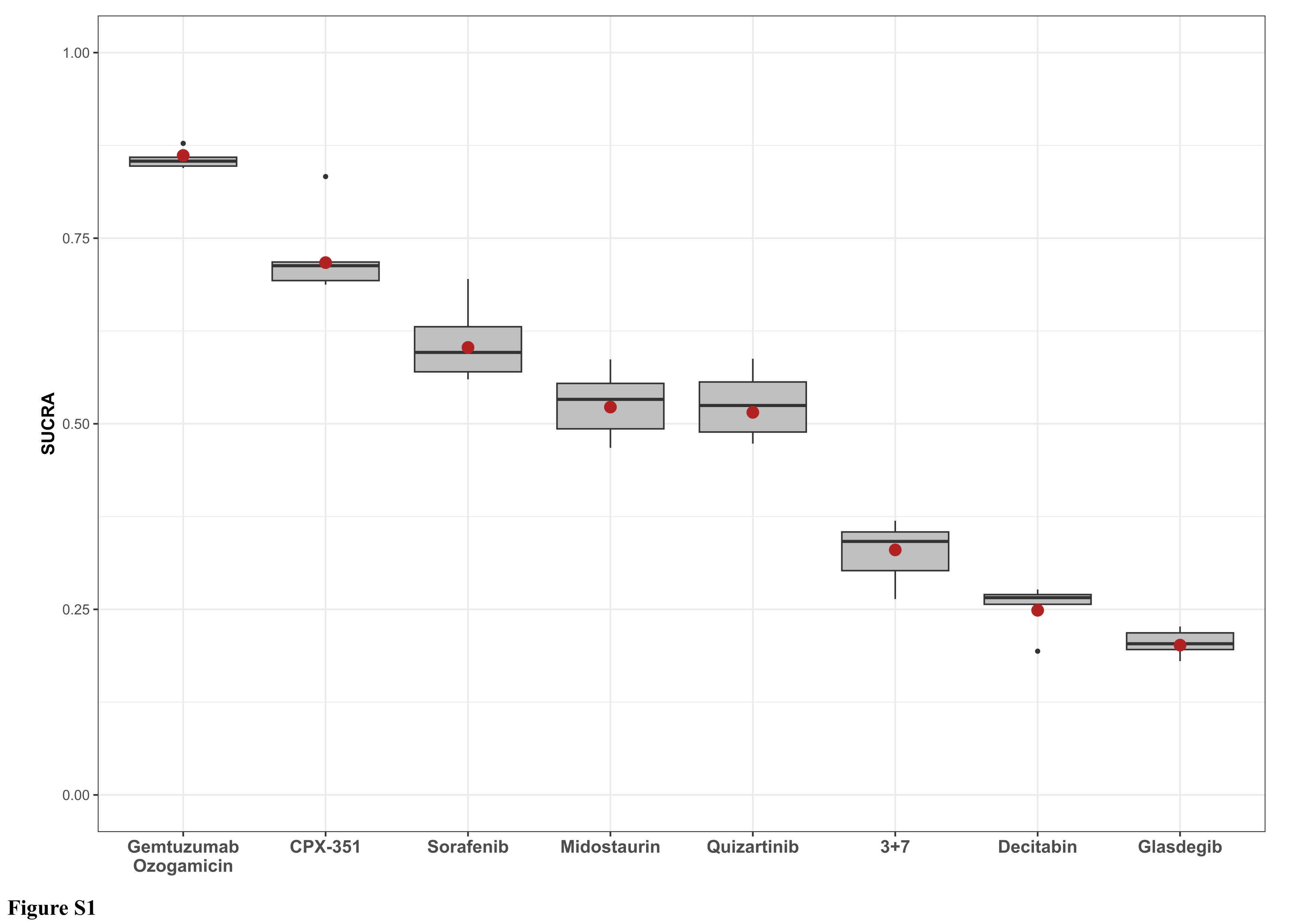

Supplement: Supplementary file 3 — (TIFF 257 KB) [file 277_2026_6948_MOESM2_ESM.tiff]

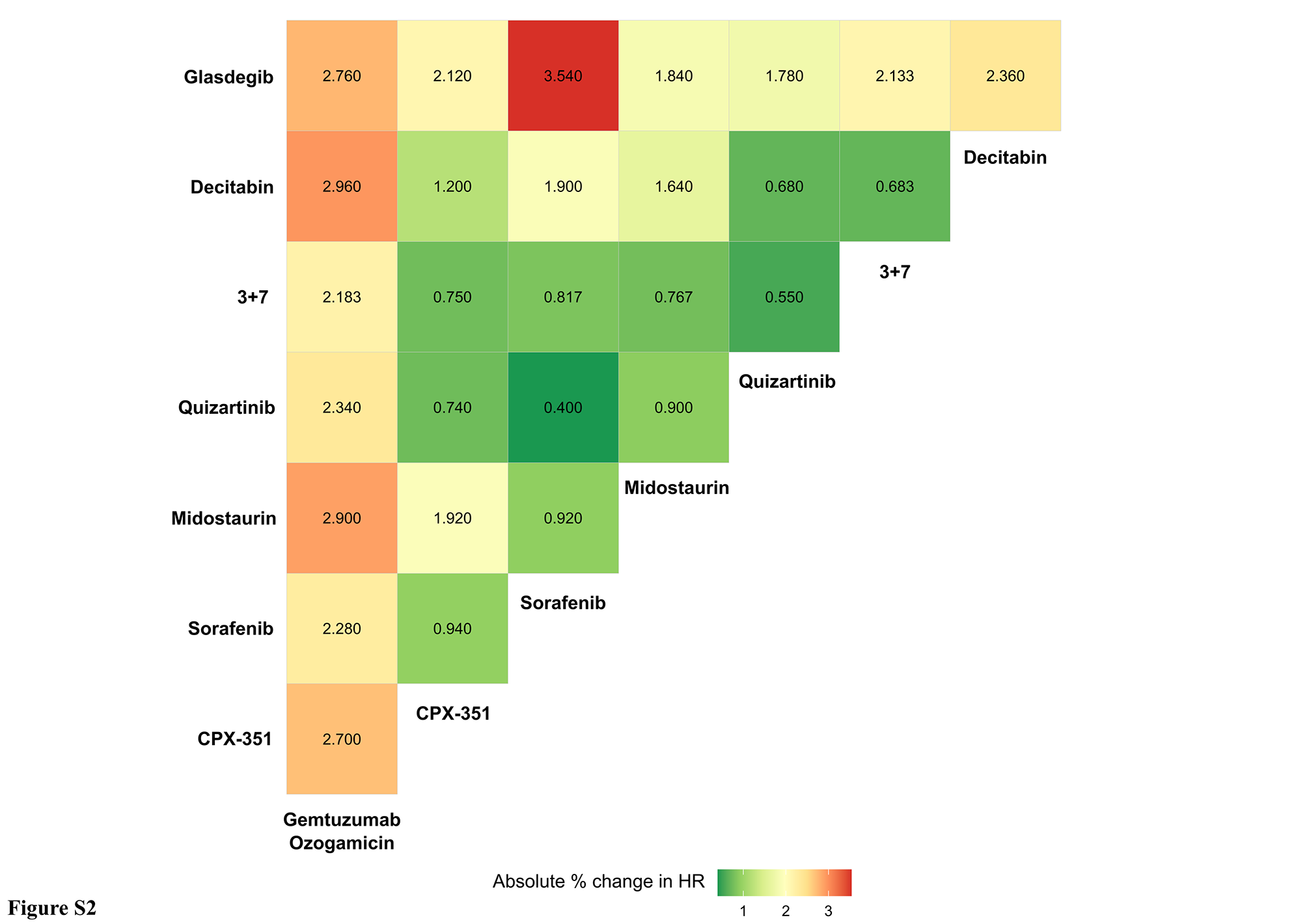

Supplement: Supplementary file 4 — (PNG 150 KB) [file 277_2026_6948_Fig5_ESM.png]

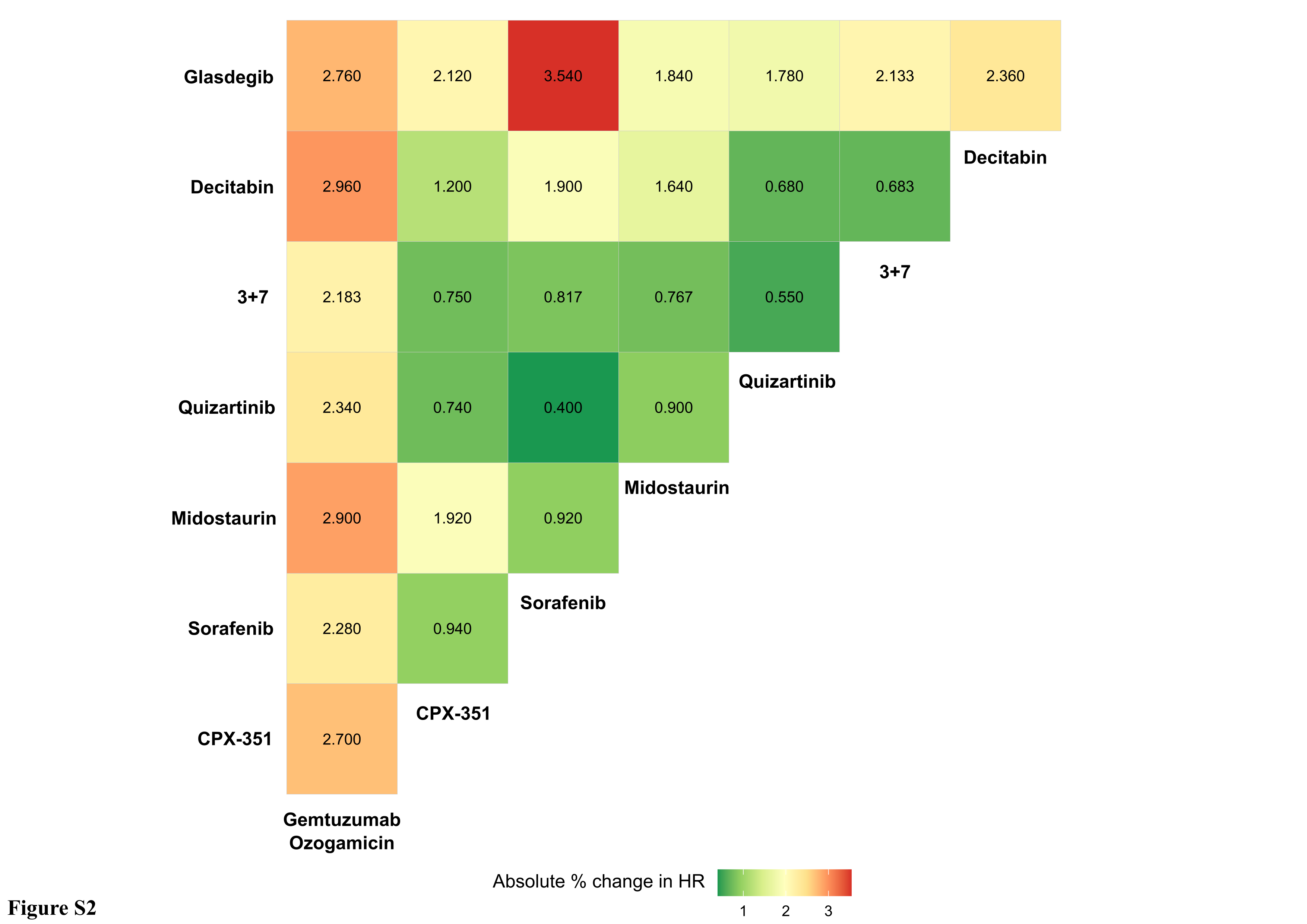

Supplement: Supplementary file 5 — Supplementary file3 (TIFF 369 KB) [file 277_2026_6948_MOESM3_ESM.tiff]
